# Study on the Numerical Simulation of the SLM Molten Pool Dynamic Behavior of a Nickel-Based Superalloy on the Workpiece Scale

**DOI:** 10.3390/ma12142272

**Published:** 2019-07-15

**Authors:** Liu Cao, Xuefeng Yuan

**Affiliations:** Advanced Institute of Engineering Science for Intelligent Manufacturing, Guangzhou University, Guangzhou 510006, China

**Keywords:** selective laser melting, molten pool dynamic behavior, equivalent processing model, workpiece scale, nickel-based superalloy, numerical simulation

## Abstract

Nickel-based superalloys are one of the most industrially important families of metallic alloys at present. Selective Laser Melting (SLM), as one of the additive manufacturing technologies for directly forming complex metal parts, has been applied in the production of Inconel 718 components. Based on the more reasonable and comprehensive equivalent processing models (vaporization heat loss, equivalent physical parameters) for the nickel-based superalloy SLM process, an SLM molten pool dynamic behavior prediction model on the workpiece scale was established. Related equivalent processing models were customized by secondary development with the software Fluent. In order to verify the feasibility of the SLM molten pool dynamics model, the SLM single-pass employed to form the Inconel 718 alloy process was calculated. The simulated and experimental solidified track dimensions were in good agreement. Then, the influences of different process parameters (laser power, scanning speed) on the SLM formation of the Inconel 718 alloy were calculated and analyzed. The simulation and experimental solidified track widths were well-matched, and the result showed that, as a rule, the solidified track width increased linearly with the laser power and decreased linearly with the scanning speed. This paper will help lay the foundation for a subsequent numerical simulation study of the thermal-melt-stress evolution process of an SLM workpiece.

## 1. Introduction

Superalloys are suitable for long-term operation in high-temperature environments and meet corrosion and abrasion requirements. They are the key metal structural materials in today’s aerospace, power, and defense fields [1,2]. Among them, the Inconel 718 nickel-based superalloy is one of the most industrially important families of metallic alloys at present, due to its excellent comprehensive properties, and is widely used in many products, including aircraft engine turbine disks, fasteners, and blades [3,4]. However, with the continuous industrial demand for improvement, traditional Inconel 718 alloy smelting, forging, and reduced material processing methods have gradually made it difficult to meet the growing processing requirements for complex parts. Based on this idea, “layer by layer” additive manufacturing technology can directly and precisely manufacture digital models into three-dimensional solid parts with a high flexibility, no mold, and no restrictions on the part structure [5]. Selective Laser Melting (SLM), as one of the additive manufacturing technologies for directly forming complex metal parts, has been applied in aerospace, automotive, medical, and other fields [6].

At present, the research on SLM formed metal parts mainly relies on experimental means. The research directions include the SLM formation mechanism, the influence of process parameters on the quality of parts, and generating the formation process in situ [7,8]. Kruth et al. [9] found, through experiments, that the effects of the laser on the SLM process were mainly reflected in three aspects: laser wavelength, energy density, and laser mode. Strano et al. [10] presented an investigation of the surface roughness and morphology for SLM parts, and the surface analysis showed an increasing density of spare particles positioned along the step edges as the surface sloping angle increased. A new mathematical model was developed to include the presence of particles on top surfaces, in addition to the stair step effect, for the accurate prediction of surface roughness. Liu et al. [11] investigated the influences of scanning speed, powder thickness, and laser power on the formation of a nickel-based superalloy by SLM, and the results showed that the synergistic effects of laser power and laser scanning speed affected the formation quality. However, the complex thermophysical interactions that existed during the SLM process often occurred on a very short, microsecond time scale. Among them, the thermodynamics and dynamics evolution mechanisms made it difficult to achieve good analytical results through engineering experiments, which restricted the essential understanding of the problems of microstructure control, internal defect formation, deformation, and cracking of the workpiece during the current SLM engineering application process. The method of numerical simulation has been widely used in industrial production for its forward-thinking nature and has been applied in studying physical processes and preventing defects in mechanical manufacturing [12,13].

In the past ten years, numerical simulation studies on the SLM forming process have gradually emerged [14,15], and these theoretical research works can be roughly divided into two directions: based on the particle scale [16,17] and based on the workpiece scale [18,19].

### 1.1. Numerical Simulation of SLM Molten Pool Dynamic Behavior on the Particle Scale

The so-called particle scale refers to the modeling based on the actual particle morphology, directly calculating the heating and melting effects of the laser on the metal particles, and then describing the complex flow behavior of the metal liquid between the particles on the order of micrometers. Voisin et al. [20] used the multi-physics code ALE3D to study the dynamic behavior of the SLM molten pool based on the particle scale, and directly calculated the distribution of pore defects at different scanning speeds. Lee et al. [21] used the open source discrete element method (DEM) code Yade to obtain the initial distribution of laminated particles, and used the commercial software Flow-3D to calculate the SLM single pass process to study the formation of a ball defect through the simulation results. Panwisawas et al. [22] carried out a numerical simulation of the dynamic behavior of the SLM molten pool based on the open source computational fluid dynamics (CFD) code OpenFOAM, and compared the effects of different lamination thicknesses on the formation effects. This kind of simulation method can directly describe the SLM formation process and directly predict the formation and evolution of defects, such as pores and balls, but the calculation requirements are often huge (the number of elements is tens of millions, and the required computing resources reach the order of 10^5^ cpu.hrs). It needs to be implemented with a supercomputer, and the calculation size is often limited to a few hundred microns.

### 1.2. Numerical Simulation of SLM Molten Pool Dynamic Behavior on the Workpiece Scale

The so-called workpiece scale refers to the powder layer (including metal particles and pores) as a special material, indirectly describing the temperature and flow field evolution in the SLM forming process by setting equivalent physical parameters and flow behavior models, where the mesh size is often a few hundred microns, or even a few millimeters. The reason for the higher computational efficiency of this method is that there is no need to describe the movement of the pores inside the powder bed. Xiao et al. [23] used the idea of a continuous medium (single phase with a uniform material distribution) to calculate the shape of the molten pool during the SLM process and considered the influence of buoyancy and the Marangoni effect on the internal flow behavior of the molten pool, but did not consider vaporization heat loss. Gusarov et al. [24] proposed utilizing equivalent thermal conductivity to characterize the thermal conduction of the powder layer. The equivalent radiation heat transfer model was used to calculate the heating effect of the laser beam on the powder layer, and the influence of the laser beam mode on the SLM process was studied. Yuan et al. [25] carried out a numerical simulation of the SLM process using Fluent, analyzed the internal flow of the molten pool caused by the Marangoni effect, and compared the influence of different process parameters on the size of the molten pool. This kind of simulation method cannot describe the SLM formation process intuitively, but the advantage is that the temperature, flow, and stress field evolution in the SLM process can be described by equivalent processing methods, and then the temperature, the shape of the molten pool, and the deformation of the workpiece during the entire formation process can be obtained. Due to the unusual complexity of the SLM formation process, the accuracy of the simulation method based on the workpiece scale mainly depends on the rationality of the equivalent processing models.

In summary, the calculation efficiency of the research method based on the particle size was too low, which makes it difficult to quickly predict and analyze the SLM process. Therefore, the research method based on the workpiece scale was selected. However, due to the incompleteness of the equivalent processing methods currently used in the research based on the workpiece scale, the calculation accuracy was low. In this paper, by introducing more reasonable and comprehensive equivalent processing models (vaporization heat loss, equivalent physical parameters), a dynamic behavior prediction model of an SLM molten pool based on the workpiece scale has been established for the nickel-based superalloy SLM process. The secondary development method was used to customize the relevant equivalent processing models based on Fluent, and a numerical simulation of the SLM formation process of a nickel-based superalloy was carried out. To verify the feasibility of the SLM molten pool dynamics model, the SLM single-pass formation of the Inconel 718 alloy was calculated and compared to the experimentally obtained solidified track size. Then, the influences of different process parameters (laser power, scanning speed) on the SLM formation of the Inconel 718 alloy were analyzed, and the calculation results were verified with the experimental results. This study can be expected to help lay the foundation for a subsequent numerical simulation study of the thermal-melt-stress evolution process of SLM parts.

## 2. Mathematical and Numerical Modeling

### 2.1. Dynamic Behavior Control Equations of the SLM Molten Pool Based on the Workpiece Scale

In a study of the dynamic behavior of the SLM molten pool based on the workpiece scale, the calculation area consists of four parts: the powder bed, the solidified portion, the metal base plate, and the protective atmosphere chamber. In the calculation process, the powder bed is gradually transformed into the solidified portion by the equivalent treatment, and energy and momentum interactions occur between the parts. In addition, in order to ensure the efficiency of the numerical calculation, several appropriate assumptions have been made, including: not considering the mass loss caused by the vaporization of molten metal; not considering the influence of the change in metal density on the volume; and considering that the fluids involved in the calculation are all incompressible, Newtonian fluids. These assumptions mean that the mass of the metal phase in the calculation was constant, the influence of the volume change of the metal phase on the flow behavior was not considered, and the compressibility of the gas phase and the liquid metal was not considered. Next, the three types of conservation equations used in this study will be introduced.

#### 2.1.1. Momentum Conservation Equation

When metal particles are melted by laser radiation, factors affecting the flow behavior of the liquid metal include: surface tension between the liquid metal and the substrate and particles, the Marangoni effect (surface tension gradient caused by the temperature difference on the liquid metal surface), the vaporization recoil force of the liquid metal, buoyancy, the internal pressure of the liquid metal, the internal viscous force of the liquid metal, gravity, and the difference in fluidity between the liquid and solid metal during solidification. Among them, the first three influencing factors are surface forces and the last five influencing factors are volumetric forces. Since the calculation model used here is a single-phase flow model (the coupling between gas phase and liquid metal was not calculated) and considering that vaporization recoil force mainly affects the liquid surface fluctuation of liquid metal, this study does not model these factors. The obtained momentum conservation equation is as below.

(1) $$\frac{\partial \rho u}{\partial t}+\nabla \cdot \left(\rho u⊗u\right)=-\nabla p+\nabla \cdot \bar{\tau }+\rho g+F_{buoyancy}+F_{mushy}$$

where

(2) $$\bar{\tau }=2\mu \left[\left(\frac{1}{2}\nabla u+\frac{1}{2}{\left(\nabla u\right)}^{T}\right)-\frac{1}{3}\left(\nabla \cdot u\right)I\right]$$

(3) $$F_{buoyancy}=\rho g\beta \left(T-T_{ref}\right)$$

(4) $$F_{mushy}=-\rho K_{C}\left[\frac{{\left(1-f_{l}\right)}^{2}}{f_{l}^{3}+C_{K}}\right]u$$

Here, ρ is the density, kg/m^3^; u is the velocity, m/s; t is the time, s; ⊗ is the tensor product; p is the pressure, Pa; $\bar{\tau }$ is the stress tensor; g is the gravity acceleration, m/s^2^; $F_{buoyancy}$ is the buoyancy, N/m^3^; $F_{mushy}$ is the mushy zone drag force, which can be used to characterize the difference in fluidity caused by the liquid-solid transition [26], N/m^3^; μ is the dynamic viscosity, Pa·s; I is the unit matrix; β is the thermal expansion coefficient, 1/K; T is the temperature, K; $T_{ref}$ is the thermal expansion reference temperature, K; $K_{C}$ is the porous media permeability coefficient, 1/s; $C_{K}$ is a custom smaller value, which is used to avoid the drag force of the mushy zone during the calculation to infinity; and $f_{l}$ is the liquid fraction of the metal phase.

The right-end terms in Equation (1) characterize the five-volume forces (internal pressure, internal viscous force, gravity, buoyancy, and mushy zone drag force) experienced by the liquid metal, respectively. Because the laser energy density is Gaussian in the horizontal plane, the liquid metal surface temperature shows a central high and a peripheral low, and since the surface tension is related to temperature, the tangential flow on the liquid surface occurs under the influence of the surface tension gradient, so the Marangoni effect needs to be characterized by defining the corresponding surface force. The boundary condition used to describe the Marangoni effect [27] here is

(5) $$-\mu \frac{\partial u_{x}}{\partial z}=\frac{d\sigma }{dT}\frac{\partial T}{\partial x}$$

(6) $$-\mu \frac{\partial u_{y}}{\partial z}=\frac{d\sigma }{dT}\frac{\partial T}{\partial y}$$

Here, $\frac{d\sigma }{dT}$ is the surface tension coefficient with the rate of change in temperature, N/(m·K); x, y are the coordinates of the horizontal plane, m; z is the coordinate in the vertical direction, m; and $u_{x},\;u_{y}$ are the components of the tangential velocity on the liquid metal surface, m/s.

#### 2.1.2. Energy Conservation Equation

The factors to be considered in the calculation of the temperature field of the SLM process include the absorption of laser energy, melting of the solid metal, vaporization of the liquid metal, convection diffusion inside the metal phase, and heat exchange between the metal phase and the surroundings (convection and radiation). The adopted energy conservation equation is

(7) $$\frac{\partial \rho c_{e}T}{\partial t}+\nabla \cdot \left(\rho uc_{e}T\right)=\nabla \cdot \left(k\nabla T\right)+Q_{laser}$$

where

(8) $$c_{e}=\{\begin{array}{ll} c+\frac{L_{f}}{T_{l}-T_{s}} & T_{l}<T<T_{s}\\ c & T\geq T_{l}\;or\;T\leq T_{s} \end{array}$$

Here, $c,\;c_{e}$ represent the specific heat capacity of the metal phase and the equivalent specific heat capacity [28], respectively, J/(kg·K); k is the thermal conductivity, W/(m·K); $Q_{laser}$ is the laser energy density, W/m^3^; $L_{f}$ is the metal melting latent heat, J/kg; and $T_{l},\;T_{s}$ are the metal liquidus and solidus temperatures, respectively, K.

Since the vaporization heat loss of the liquid metal and the heat exchange between the metal phase and the surroundings are carried out through the surface, the heat transfer boundary condition used is

(9) $$q_{transfer}=-q_{con}-q_{rad}-q_{vap}$$

where

(10) $$q_{con}=h_{c}\left(T-T_{con}\right)$$

(11) $$q_{rad}=\sigma_{s}\varepsilon \left(T^{4}-T_{rad}^{4}\right)$$

Here, $q_{transfer},\;q_{con},\;q_{rad},\;q_{vap}$ are the total heat exchange, convective heat transfer, radiation heat transfer, and vaporization heat loss, respectively, W/m^2^; $h_{c}$ is the convective heat transfer coefficient, W/(m^2^·K); $T_{con}$ is the convection temperature of the surroundings, K; $\sigma_{s}$ is the Stefan–Boltzmann constant, W/(m^2^·K^4^); ε is the emissivity; and $T_{rad}$ is the radiative temperature of the surroundings, K.

In addition, the equivalent physical property parameters (to describe the transition of the powder layer to the solidified portion), the laser energy density $Q_{laser}$, and the vaporization heat loss $q_{vap}$ will be separately described later.

#### 2.1.3. Mass Conservation Equation

Since the fluids involved are considered incompressible fluids in the calculation process, the mass conservation equation is

(12) ∇⋅u=0

### 2.2. Gaussian Body Heat Source Considering Laser Reflection between Particles

Unlike the heat source in the welding process, a laser beam will be reflected multiple times between particles during the SLM formation process [29], so the laser can be considered to heat the particles at different positions (especially in the height direction) almost simultaneously. Therefore, the heat model needs to describe the reflection process of the laser beam between the particles. However, due to the simulation study being based on the workpiece scale, the powder layer is regarded as a special material, so the surface heat source or the body heat source can only be used to characterize the energy propagation of the laser.

**Figure 1:**
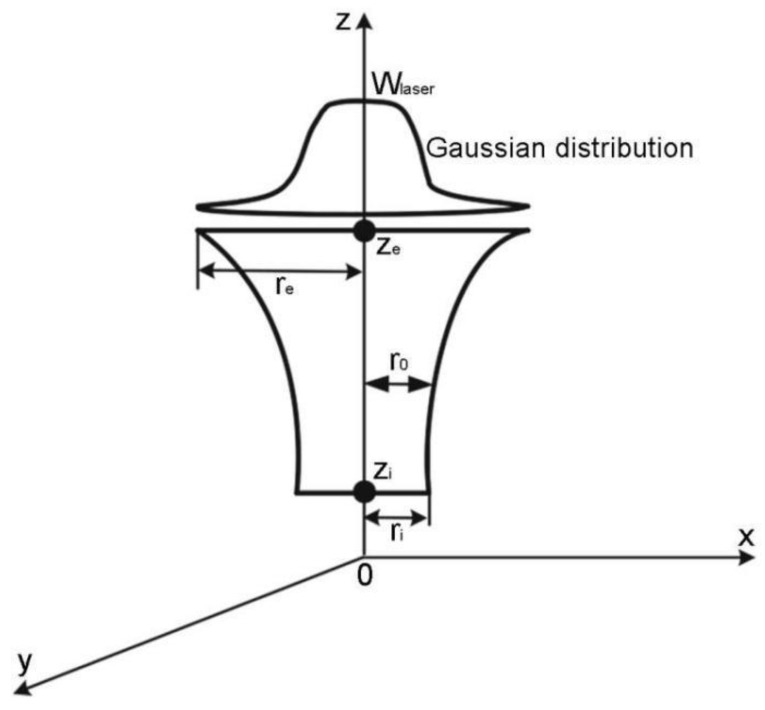
Energy density distribution of the Gaussian body heat source.

The laser energy model used here is a Gaussian body heat source [30]. The energy density in the cross-section of the heat source model is Gaussian, and the energy density in the height direction considers the difference in energy density between the upper and lower end faces caused by laser reflection. Figure 1 is the schematic of the energy density distribution of the body heat source. The mathematical expression of the heat source model is

(13) $$Q_{laser}=\frac{\xi \eta W_{laser}}{\pi \left(1-e^{-3}\right)\left(E+F\right)}\left(\frac{1-\chi }{z_{e}-z_{i}}z+\frac{\chi z_{e}-z_{i}}{z_{e}-z_{i}}\right)\exp\left(-\frac{3r^{2}}{r_{0}^{2}}\right)$$

where

(14) $$r_{0}=\frac{z^{2}}{w}+s$$

(15) $$w=\frac{z_{e}^{2}-z_{i}^{2}}{r_{e}-r_{i}}$$

(16) $$s=\frac{r_{i}z_{e}^{2}-r_{e}z_{i}^{2}}{z_{e}^{2}-z_{i}^{2}}$$

(17) $$E=\frac{1-\chi }{z_{e}-z_{i}}\left\{\left(\frac{1}{w^{2}}\frac{z_{e}^{6}}{6}+\frac{s}{w}\frac{z_{e}^{4}}{2}+\frac{s^{2}}{2}z_{e}^{2}\right)-\left(\frac{1}{w^{2}}\frac{z_{i}^{6}}{6}+\frac{s}{w}\frac{z_{i}^{4}}{2}+\frac{s^{2}}{2}z_{i}^{2}\right)\right\}$$

(18) $$F=\frac{\chi z_{e}-z_{i}}{z_{e}-z_{i}}\left\{\left(\frac{1}{w^{2}}\frac{z_{e}^{5}}{5}+2\frac{s}{w}\frac{z_{e}^{3}}{3}+s^{2}z_{e}\right)-\left(\frac{1}{w^{2}}\frac{z_{i}^{5}}{5}+2\frac{s}{w}\frac{z_{i}^{3}}{3}+s^{2}z_{i}\right)\right\}$$

Here, $W_{laser}$ is the laser power, W; ξ is the energy distribution factor; η is the effective absorption factor; χ is the ratio of the central energy density of the lower end face to the upper end face; $z_{e},\;z_{i}$ are the height coordinates of the upper and lower end faces of the laser energy distribution area, respectively, m; $r_{e},\;r_{i}$ are the radii of the upper and lower end faces of the laser energy distribution area, respectively, m; $r_{0}$ is the laser distribution cross-section radius when the height coordinate is z, m; and w, s, E, F are calculated intermediates.

### 2.3. Vaporization Heat Loss Model

For general metals, the vaporization temperature is around 3000 K. The laser beam has a very high energy density during the SLM process, and it is often able to vaporize the metal in a very short time. Therefore, an accurate SLM numerical simulation needs to consider the effects of vaporization heat loss and the vaporization recoil force. The vaporization heat loss model [31] used here is

(19) $$q_{vap}={\hat{m}}_{vap}\Delta H_{vap}$$

where

(20) $${\hat{m}}_{vap}=\left(p_{vap}-p_{amb}\right)\sqrt{\frac{m}{2\pi k_{B}T}}$$

Here, ${\hat{m}}_{vap}$ is the mass of the vaporized liquid metal on a unit of liquid surface per unit time, kg/(m^2^·s); $\Delta H_{vap}$ is the metal vaporization latent heat, J/kg; m is the metal molecular mass of the metal, kg; $k_{B}$ is the Boltzmann constant, J/K; $p_{vap}$ is the vaporization pressure, Pa; and $p_{amb}$ is the protective atmosphere pressure, Pa.

The calculation of $p_{vap}$ uses the vaporization pressure model under different environmental pressures, as proposed by Pang et al. [32]:

(21) $$p_{vap}=\{\begin{array}{ll} p_{amb} & 0\leq T<T_{left}\\ \frac{1+\beta_{r}}{2}p_{0}\exp\left[\frac{m\Delta H_{vap}}{k_{B}}\left(\frac{1}{T_{v}}-\frac{1}{T}\right)\right] & T\geq T_{right}\\ p_{smooth} & T_{left}\leq T<T_{right} \end{array}$$

Here, $p_{0}$ is the standard atmospheric pressure, Pa; $T_{v}$ is the metal vaporization temperature, K; $T_{left},\;T_{right}$ are the left and right critical temperatures of the transition zone, respectively, K; $p_{smooth}$ is the transition zone pressure, Pa; and $\beta_{r}$ is the recombination rate, and its value depends on the Mach number of the vapor plume. For high gasification rate conditions (such as a vacuum or at a high laser intensity), $\beta_{r}=0.18$, and for low gasification rate conditions (such as a high ambient pressure or at a low laser intensity), $\beta_{r}=1$. In other cases, the value of $\beta_{r}$ is between the two.

**Figure 2:**
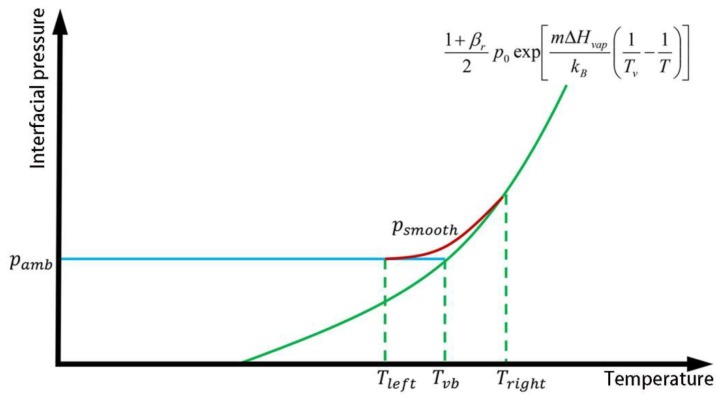
Schematic of the vaporization pressure model.

The effect of the transition zone pressure, $p_{smooth}$, is employed to achieve a smooth interfacial pressure over the entire temperature range (Figure 2). The junction temperature, $T_{vb}$, in Figure 2 can be calculated by the following formula:

(22) $$\frac{1+\beta_{r}}{2}p_{0}\exp\left[\frac{m\Delta H_{vap}}{k_{B}}\left(\frac{1}{T_{v}}-\frac{1}{T_{vb}}\right)\right]=p_{amb}$$

The left and right critical temperatures of the transition zone, $T_{left},\;T_{right}$, satisfy the following (the coefficient of 0.05 is an artificially set value for smoothing):

(23) $$T_{right}-T_{vb}=T_{vb}-T_{left}=0.05T_{vb}$$

The transition zone pressure, $p_{smooth}$, can be defined as

(24) $$p_{smooth}=aT^{3}+bT^{2}+cT+d$$

In order to ensure a smooth transition of the interface pressure at $T_{left}$ and $T_{right}$, the distribution of $p_{smooth}$ can be obtained according to the coordinates of the two ends and the slopes of the tangents.

### 2.4. Equivalent Thermal Property Parameters Based on the Formation State

The core aim of the numerical simulation based on the workpiece scale is to equate the powder layer to a special material, but, in the actual SLM process, the powder layer will undergo a process of melting into liquid metal and become a dense solid. Therefore, it can be considered that the powder layer undergoes three state transitions: a particle state, a liquid state, and a solid state. In the calculation process, the basis for judging whether the state of the powder layer is changed is: (1) once the temperature of the original particle element exceeds its melting temperature (generally taken as the intermediate value of the liquidus and solidus temperature), the element state is converted to a liquid state; (2) for the elements that were originally in the liquid or solid state, their state will only change between liquid and solid (based on the liquidus-solidus temperature of the metal). The equivalent physical properties (density, specific heat capacity, and thermal conductivity) based on the formation state of the powder layer will be described below.

#### 2.4.1. Equivalent Density and Specific Heat Capacity Based on the Formation State

During the calculation, the density of the powder layer element is

(25) $$\rho =\{\begin{array}{ll} \left(1-\phi \right)\rho_{m}+\phi \rho_{a} & particle\;state\\ \rho_{m} & liquid\;state\;or\;solid\;state \end{array}$$

Here, ϕ is the initial porosity of the powder layer and $\rho_{m},\;\rho_{a}$ are the densities of the metal and gas phases, respectively, kg/m^3^. It should be noted that $\rho_{m}$ and $\rho_{a}$ are temperature-dependent. In addition, the powder layer element was treated equivalently to the specific heat capacity.

#### 2.4.2. Equivalent Thermal Conductivity Based on the Forming State

For the thermal conductivity of the powder layer, a treatment like that in Equation (25) cannot be performed (the equivalent physical property parameter is the weighted average of the physical parameters of the constituent phase). This is because, for the powder layer element in the particle state, the thermal conductivity is mainly determined by the heat conduction of the gas phase between the particles, but is also slightly affected by the thermal conductivity of the particles themselves. The equivalent thermal conductivity model [33] of the powder layer used here is

(26) $$k=\{\begin{array}{ll} \left(1-\sqrt{1-\phi }\right)\left(k_{a}+\phi k_{r}\right)+\sqrt{1-\phi }\left\{\frac{2}{\frac{1}{k_{a}}-\frac{1}{k_{m}}}\left[\frac{1}{1-\frac{k_{a}}{k_{m}}}\ln\left(\frac{k_{m}}{k_{a}}\right)-1\right]+k_{r}\right\} & particle\;state\\ k_{m} & liquid\;state\;or\;solid\;state \end{array}$$

where

(27) $$k_{r}=4F_{view}\sigma_{s}T_{P}^{3}D_{P}$$

Here, $k_{m},\;k_{a},\;k_{r}$ are the thermal conductivities of the metal phase, the protective gas, and the internal radiation of the powder layer, respectively, W/(m·K); $F_{view}$ is the internal radiation factor, which is 1/3; $T_{P}$ is the temperature of the metal particle, K; and $D_{P}$ is the average particle diameter, m. It should be noted that $k_{m}$ and $k_{a}$ are temperature-dependent.

### 2.5. Numerical Solution of the Dynamic Behavior of the SLM Molten Pool Based on the Workpiece Scale

**Figure 3:**
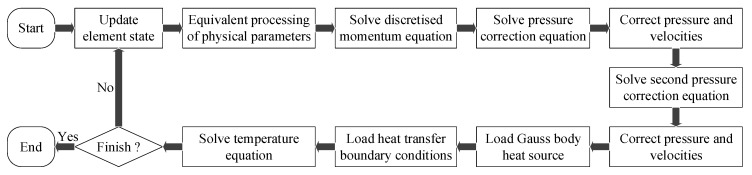
Calculation flow chart.

Based on the commercial CFD software Fluent v19.1, the numerical calculation of the dynamic behavior of the SLM molten pool on the workpiece scale was carried out. Among them, the selected solution models were Energy, Viscous-Laminar, and Solidification & Melting. User Defined Functions (UDFs) included a moving Gaussian body heat source, heat transfer boundary conditions (convection heat dissipation, radiation heat dissipation, and vaporization heat loss), and equivalent physical parameters (density, specific heat capacity, and thermal conductivity). The pressure–velocity coupling algorithm used SIMPLEC, and the time step was 1 ns. Figure 3 is the calculation flow chart for this study.

## 3. Results and Discussion

According to the physical model and numerical solution described above, the dynamic behavior of the SLM molten pool on the workpiece scale was predicted by using Fluent. First, in order to verify the feasibility of the SLM molten pool dynamics model, the single-pass process was calculated and compared to the experimentally obtained solidified track size, according to the experimental conditions for forming the Inconel 718 alloy by SLM outlined by Zhang et al. [34]. Secondly, the influences of different process parameters (laser power, scanning speed) on the SLM formation of the Inconel 718 alloy were analyzed, and the calculation results were verified with the experimental results according to the SLM experiment done by Wu et al. [35]. The mesh generation tool used ICEM CFD v19.1 and CFD-Post v19.1 was used for post-processing.

### 3.1. Experimental Verification of the Inconel 718 Nickel-Based Superalloy by the SLM Process

#### 3.1.1. Calculation Parameters and Mesh Model

The composition (mass percentage) of the Inconel 718 alloy is Ni 50.4-Fe 21.86-Cr 18.44-Nb 5.04-Mo 3.02-Ti 0.88-Al 0.33-C 0.03. Table 1 contains the Inconel 718 physical parameters calculated by JMatPro-v7.

The vaporization pressure, $p_{vap}$, Pa, of the Inconel 718 alloy was calculated according to Equations (21)–(24):

(28) $$p_{vap}=\{\begin{array}{ll} 1.01325\times 10^{5} & 0<T<2935\\ 3.376372\times 10^{-3}T^{3}-29.4454291T^{2}+85590.17272T-8.28202513\times 10^{7} & 2935\leq T<3244\\ 60795\exp\left[52724\times \left(\frac{1}{3000}-\frac{1}{T}\right)\right] & T\geq 3244 \end{array}$$

The protective atmosphere in the experiment was argon and the other parameters required for the calculation are shown in Table 2.

**Figure 4:**
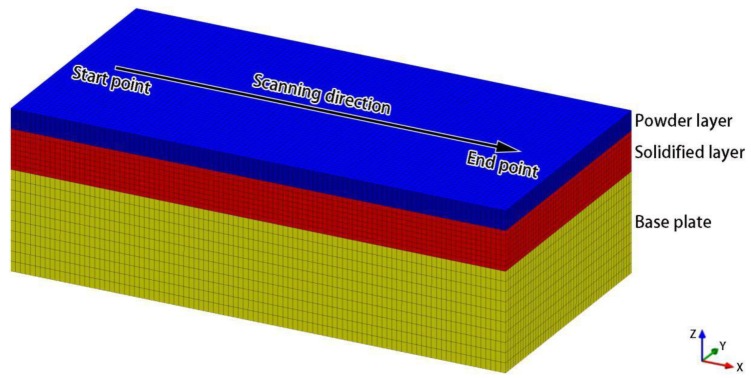
Adopted geometry and mesh models.

Figure 4 shows the geometry and mesh model used here. The calculation area was divided into three parts: the power layer, the solidified layer, and the base plate. The geometric dimensions of the three parts were 1 × 0.5 × 0.04 mm^3^, 1 × 0.5 × 0.08 mm^3^, and 1 × 0.5 × 0.2 mm^3^, and the mesh sizes were 0.01 × 0.01 × 0.0025 mm^3^, 0.01 × 0.01 × 0.01 mm^3^, and 0.01 × 0.01 × 0.02 mm^3^, respectively. The number of mesh elements obtained in each part was 80,000, 40,000, and 50,000, respectively. Boundary conditions included the top surface of the power layer set to convection, radiation, and vaporization; the bottom surface of the base plate set as convective heat transfer; the contact surface between the powder layer and the solidified layer set as a coupled wall; and the other boundary faces set as heat insulation. In addition, the start position, end position, and scanning direction of the laser in the single pass process are indicated in Figure 4 (the x coordinates of the start and end points are 0.1 mm and 0.9 mm, respectively).

#### 3.1.2. Comparison of Simulation and Experimental Solidified Track Sizes

**Figure 5:**
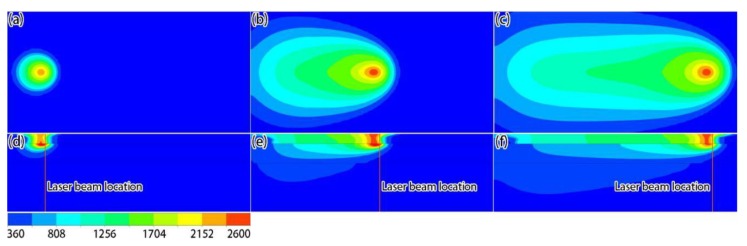
Simulation results of temperature fields in the top (**a**–**c**) and middle (**d**–**f**) sections at different times: (**a**,**d**) 6 × 10^−5^ s; (**b**,**e**) 4.5 × 10^−4^ s; (**c**,**f**) 8.4 × 10^−4^ s (unit: K).

**Figure 6:**
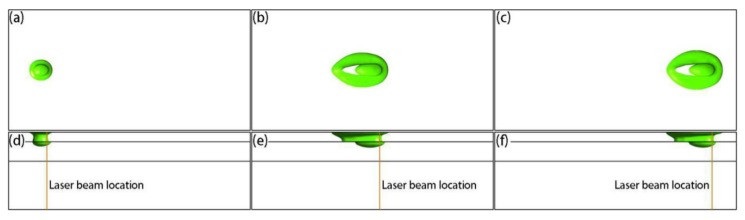
Simulation results of top- (**a**–**c**) and side- (**d**–**f**) view molten pool shapes at different times: (**a**,**d**) 6 × 10^−5^ s; (**b**,**e**) 4.5 × 10^−4^ s; (**c**,**f**) 8.4 × 10^−4^ s.

Figure 5 shows the temperature distributions in the top and middle sections at different times. It can be seen from the calculation results that, since the laser energy density is Gaussian in the horizontal plane, the temperature in the center of the active laser region was high, and the temperature was low around the periphery (Figure 5a–c). From the temperature distribution in the middle section (Figure 5d–f), the highest temperature of the pool was not at the center of the laser beam, indicating that the metal particles at the center of the laser spot were not completely melted. It can also be seen from the figure that, as the laser started to heat the powder layer, the upper surface of the solidified layer was significantly heated, meaning that there was heat exchange between the powder and the solidified layers. This heat came from two sources: part of the laser energy passing through the powder bed, and heat conduction between the powder layer and the solidified layer. Figure 6 shows the molten pool shapes at different times, which were characterized by separately extracting the liquidus temperature isothermal surface of the powder layer and the solidified layer. From the top-view (Figure 6a–c), it can be seen that, when the heat exchange in the formation process reached the quasi-steady state, the shape of the molten pool was in the shape of a teardrop. From the side-view (Figure 6d–f), it can be seen that the solidified layer was partly re-melted, due to indirect heating from the laser, which is typical for the SLM process and required to properly prepare the printed component.

**Figure 7:**
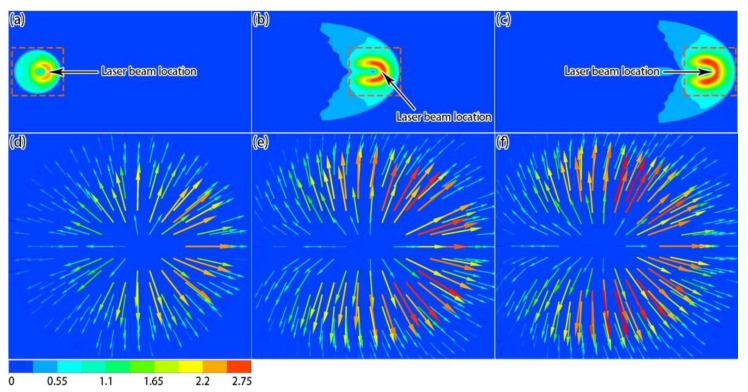
Simulation results of velocity magnitude (**a**–**c**) and local velocity (**d**–**f**) distributions on the surface at different times: (**a**,**d**) 6 × 10^−5^ s; (**b**,**e**) 4.5 × 10^−4^ s; (**c**,**f**) 8.4 × 10^−4^ s (unit: m/s).

**Figure 8:**
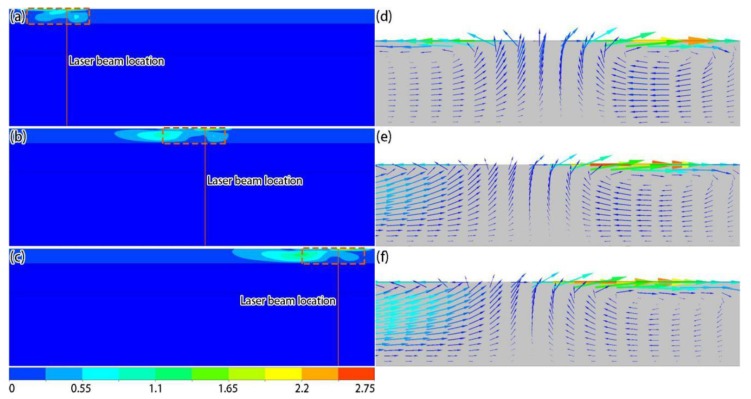
Simulation results of velocity magnitude (**a**–**c**) and local velocity (**d**–**f**) distributions in the middle section at different times: (**a**,**d**) 6 × 10^−5^ s; (**b**,**e**) 4.5 × 10^−4^ s; (**c**,**f**) 8.4 × 10^−4^ s (unit: m/s).

Figure 7 shows the velocity distributions on the top surface at different times. It can be seen from the simulation results that, due to the Gaussian distribution of the temperature on the top surface, the liquid metal flowed from the center of the molten pool to its periphery under the influence of the Marangoni effect (Figure 7d–f). Moreover, the speed distribution results (Figure 7a–c) show that the speed was low in the reverse scanning direction because the temperature of the laser-applied region was high, so the temperature gradient from the center to the activated region was low. Figure 8 shows the velocity distributions in the middle section at different times. Due to the Marangoni flow on the liquid surface, the annular convection phenomenon, centered on the molten pool axis, occurred inside the molten pool, and the tangential flow of the molten pool surface and internal convection affected the morphology of the molten pool. It should be noted that the difference in the flow behavior of the liquid and solid phases during the formation process was achieved by setting different dynamic viscosities, and a continuity condition was required in the calculation process, yielding velocity values outside of the solidified track, but their impact on the simulation results was limited.

**Figure 9:**
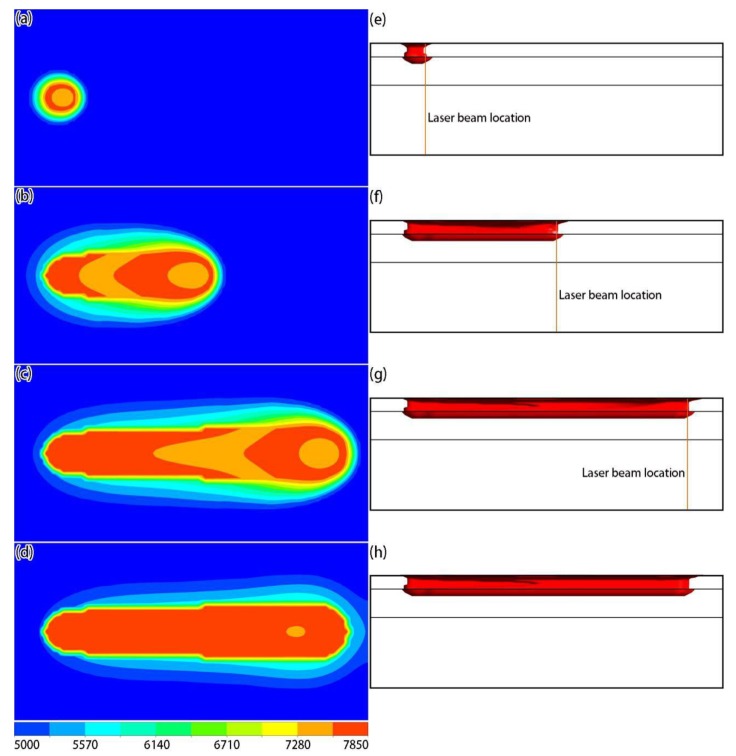
Simulation results of density distributions on the surface (**a**–**d**) and side-view solidified track shapes (**e**–**h**) at different times: (**a**,**e**) 6 × 10^−5^ s; (**b**,**f**) 4.5 × 10^−4^ s; (**c**,**g**) 8.4 × 10^−4^ s; (**d**,**h**) 1.5 × 10^−3^ s (unit: kg/m^3^).

**Figure 10:**
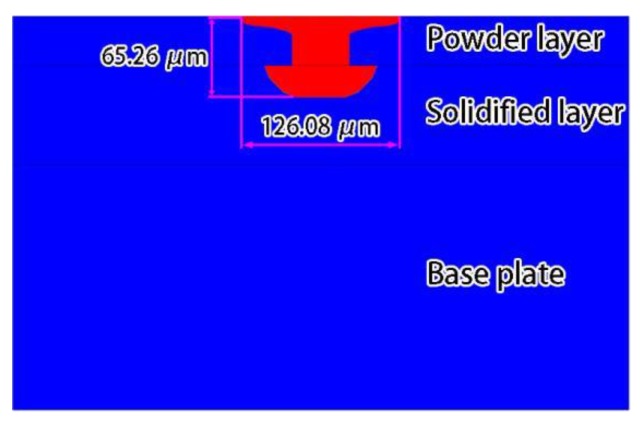
Simulation result of the transverse section of the solidified track.

Figure 9 shows the density distributions of the top surface and the side-view solidified track shapes at different times. It can be seen that the density of the powder layer element changed from the particle state to the liquid or solid state after being melted by heat, and it also reflects that the solidified track sizes were basically stable after the heat exchange reached the quasi-steady state during the formation process. Figure 10 shows the simulation result of the transverse section of the solidified track. By comparing with the experimental result [34], the experimental transverse section of the solidified track was semi-elliptical, and the powder layer and the solidified layer did not show a smooth transition to the solidified track in the simulation result. The influence of the solidified layer re-melting on the internal flow of the powder layer molten pool was not considered here, and the calculation model based on the workpiece scale could not characterize the dynamics, such as the collapse of the particles, so the temperature and velocity fields of the powder layer and the solidified layer were calculated independently. However, the key data of the SLM process was obtained through the simulation results, namely the molten pool width and depth. The simulation solidified track width was 126.08 μm and the depth was 65.26 μm (Figure 10). The experimentally obtained solidified track width was 124.14 μm and the depth was 66.21 μm, which was directly from Ref. [34]. The two agree well. Therefore, the molten pool dynamic behavior model based on the workpiece scale can be used to describe the SLM formation process to a certain extent. The model can feasibly describe the SLM process.

### 3.2. Analysis of the SLM Process of the Inconel 718 Nickel-Based Superalloy

#### 3.2.1. Calculation Parameters and Mesh Model

The parameters required to calculate this process were basically the same as those in Section 3.1.1, and Table 3 lists the different parameters. In addition, the geometric models used were nuanced. The geometric dimensions of the calculation area for the powder layer, the solidified layer, and the base plate were 1 × 0.5 × 0.03 mm^3^, 1 × 0.5 × 0.06 mm^3^, and 1 × 0.5 × 0.2 mm^3^, respectively. The corresponding mesh sizes were 0.01 × 0.01 × 0.002 mm^3^, 0.01 × 0.01 × 0.006 mm^3^, and 0.01 × 0.01 × 0.02 mm^3^. The number of mesh elements obtained in each area was 75,000, 50,000, and 50,000, respectively.

#### 3.2.2. Influence of the Laser Power on the Solidified Track Width

**Figure 11:**
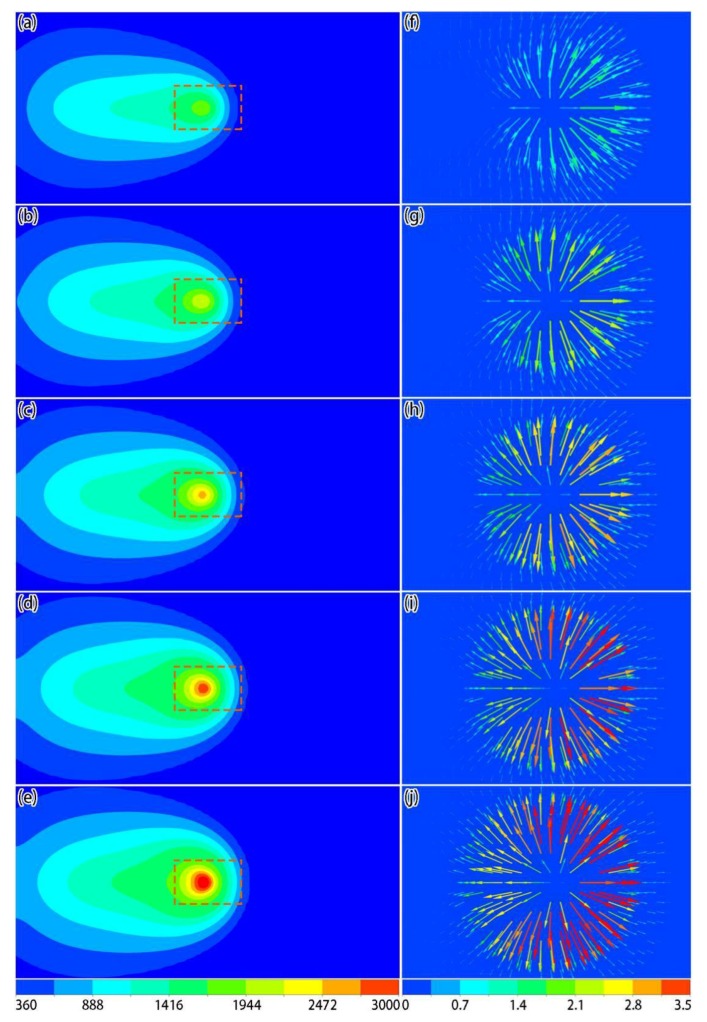
Simulation results of temperature (**a**–**e**, unit: K) and local velocity (**f**–**j**, unit: m/s) distributions on the surface under different laser powers when the laser acted on the center of the powder layer: (**a**,**f**) 150 W; (**b**,**g**) 200 W; (**c**,**h**) 250 W; (**d**,**i**) 300 W; (**e**,**j**) 350 W.

**Figure 12:**
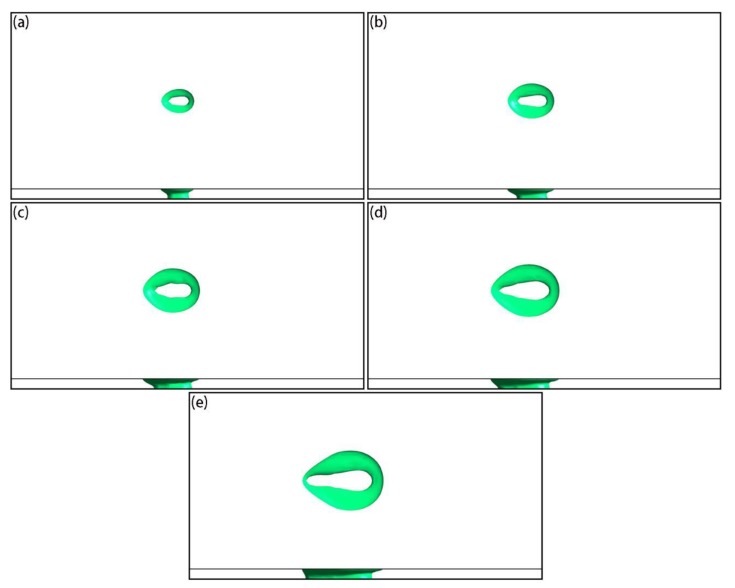
Simulation results of top- and side-view molten pool shapes under different laser powers when the laser acted on the center of the powder layer: (**a**) 150 W; (**b**) 200 W; (**c**) 250 W; (**d**) 300 W; (**e**) 350 W.

Figure 11 shows the temperature and local velocity distributions of the top surface under different laser powers when the laser acted on the center of the powder layer, where the scanning speed was set to 0.6 m/s. It can be seen that, as the laser power increased, the temperature of the active laser area increased significantly (Figure 11a–e). From the local velocity distributions (Figure 11f–j), as the laser power increased, the Marangoni effect became more apparent, and the tangential speed of the liquid metal at the surface became larger. Based on the shapes of the molten pools (Figure 12), the sizes of the molten pool also increased as the laser power increased.

**Figure 13:**
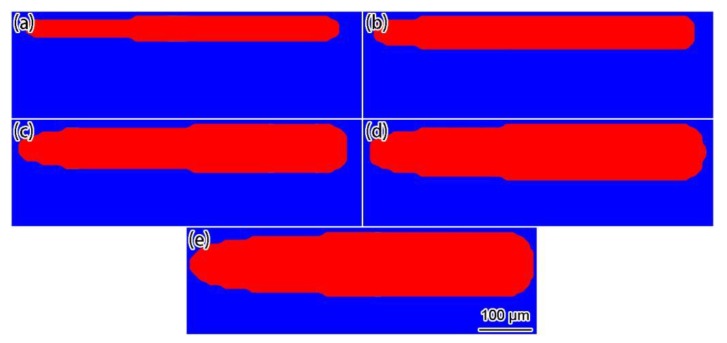
Simulation results of the final shapes of solidified tracks under different laser powers: (**a**) 150 W; (**b**) 200 W; (**c**) 250 W; (**d**) 300 W; (**e**) 350 W.

**Figure 14:**
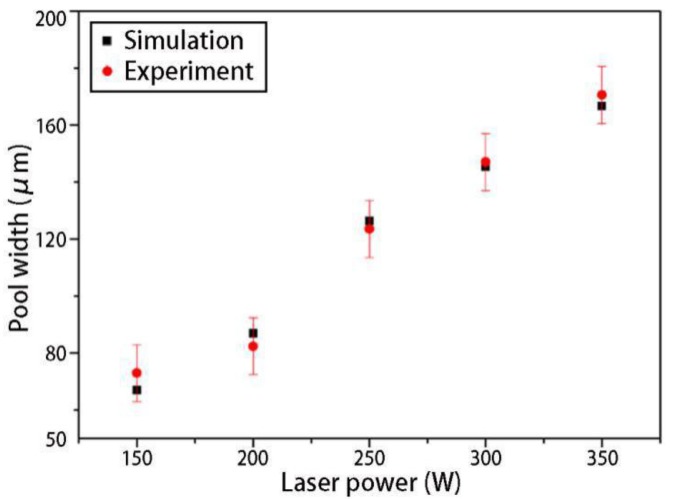
Comparison of simulation and experimental [35] solidified track widths under different laser powers.

Figure 13 shows the simulation results of the final shapes of the solidified tracks under different laser powers. From the simulation results, it can be seen that the solidified track width remained stable after the heat exchange from the SLM process reached a quasi-steady state. As the laser power increased, the width of the solidified track increased. Although the solidified track shapes in the experimental results [35] were not as regular in the simulation results, it is obvious that the solidified track width increased as the laser power increased. From the comparison of the simulation with the experimental solidified track widths under different laser powers (Figure 14, the experimental data was directly from Ref. [35]), the simulation results were in good agreement with the experimental results, and both showed that, as a rule, the solidified track width increased linearly with the laser power. It should be noted that, if the laser power was too large, the solidified track broke down due to balling and liquid instabilities [37].

#### 3.2.3. Influence of the Scanning Speed on the Solidified Track Width

**Figure 15:**
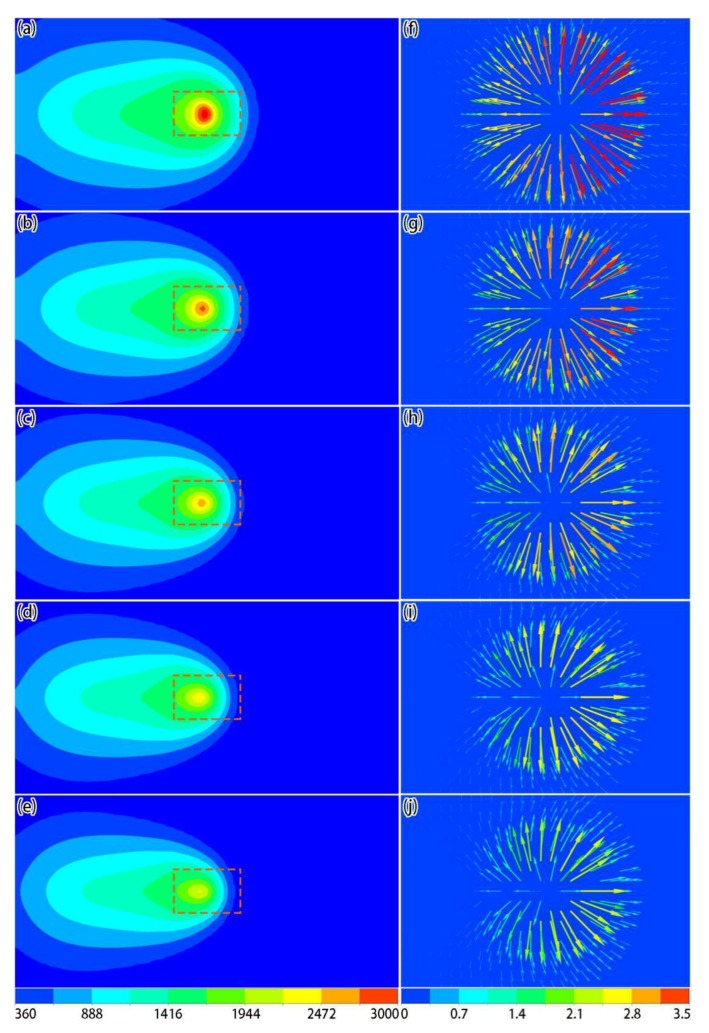
Simulation results of temperature (**a**–**e**, unit: K) and local velocity (**f**–**j**, unit: m/s) distributions on the surface under different scanning speeds when the laser acted on the center of the powder layer: (**a**,**f**) 0.4 m/s; (**b**,**g**) 0.5 m/s; (**c**,**h**) 0.6 m/s; (**d**,**i**) 0.7 m/s; (**e**,**j**) 0.8 m/s.

**Figure 16:**
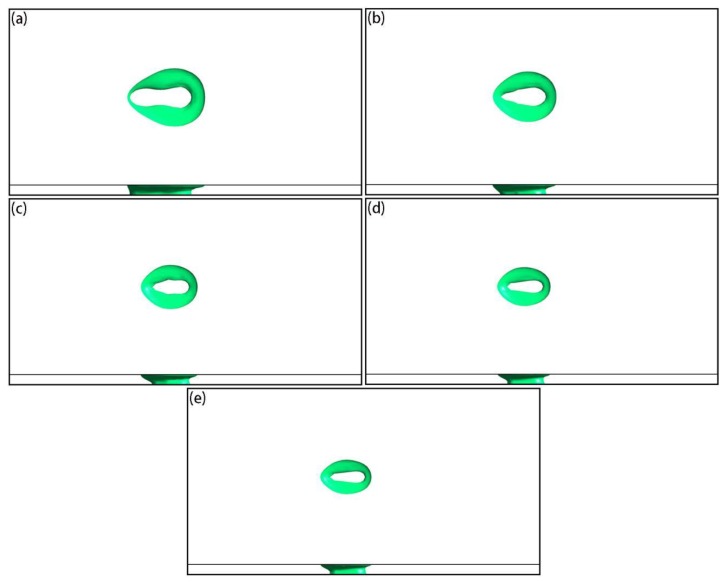
Simulation results of top- and side-view molten pool shapes under different scanning speeds when the laser acted on the center of the powder layer: (**a**) 0.4 m/s; (**b**) 0.5 m/s; (**c**) 0.6 m/s; (**d**) 0.7 m/s; (**e**) 0.8 m/s.

Figure 15 shows the temperature and local velocity distributions of the top surface under different scanning speeds when the laser acted on the center of the powder layer, where the laser power was set to 250 W. It can be seen that as the scanning speed increased, the temperature of the active laser area was significantly reduced (Figure 15a–e), because the active time of the laser on a fixed position was reduced. From the local velocity distributions (Figure 15f–j), as the scanning speed increased, the Marangoni effect weakened and the tangential speed of the liquid metal at the surface became smaller. From the shape of the molten pools (Figure 16), the sizes of the molten pool decreased as the scanning speed increased.

**Figure 17:**
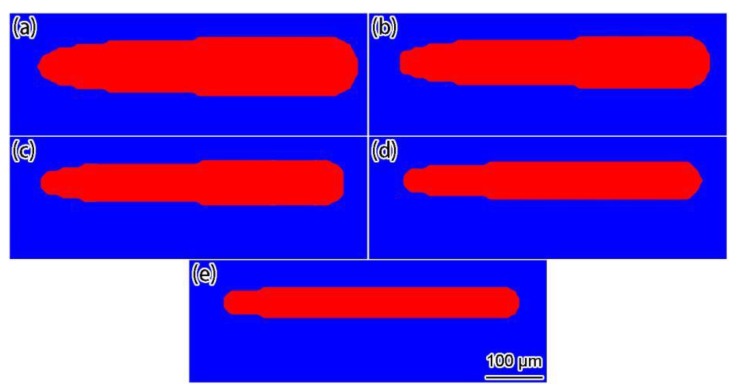
Simulation results of the final shapes of solidified tracks under different scanning speeds: (**a**) 0.4 m/s; (**b**) 0.5 m/s; (**c**) 0.6 m/s; (**d**) 0.7 m/s; (**e**) 0.8 m/s.

**Figure 18:**
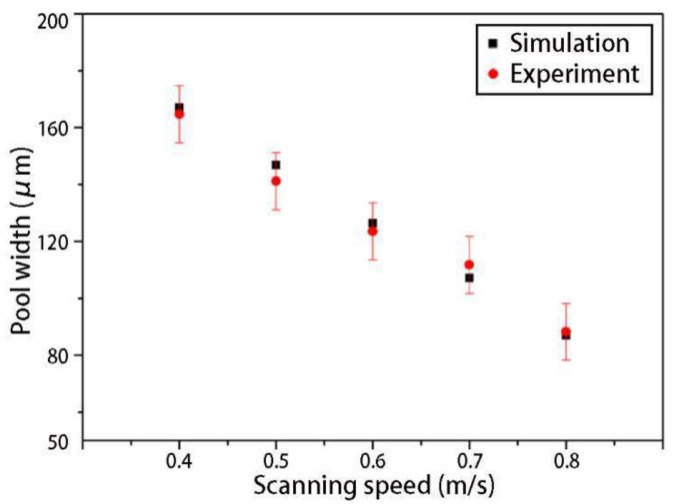
Comparison of simulation and experimental [35] solidified track widths under different scanning speeds.

Figure 17 displays the simulation results of the final shapes of the solidified tracks under different scanning speeds. From the simulation results, it can be seen that, when the heat exchange of the SLM process reached a quasi-steady state, the width of the solidified track remained stable and, as the scanning speed increased, the width of the solidified track gradually decreased. Although the solidified track shapes in the experimental results [35] were not as regular as the simulation results, it was obvious that the solidified track width decreased as the scanning speed increased. From the comparison of the simulation and the experimental solidified track widths under different scanning speeds (Figure 18, the experimental data was directly from Ref. [35]), the simulation results were in good agreement with the experimental results, and both showed, as a rule, that the solidified track width decreased linearly with the scanning speed. It should be noted that, if the scanning speed was too small, the solidified track broke down due to balling and liquid instabilities [37].

## 4. Conclusions

(1) The more reasonable and comprehensive equivalent processing models included the following. Based on the smooth vaporization pressure model, the liquid metal vaporization heat loss models were established. To characterize the transformation of the powder layer state (particle state, liquid state and solid state) in the SLM process, the equivalent density, specific heat capacity, and thermal conductivity models based on the formation state were established.
(2) The SLM single-pass formation of the Inconel 718 alloy process was calculated. The simulation and experimental solidified track sizes were in good agreement, and the feasibility of the SLM molten pool dynamics model was verified.
(3) The influences of different process parameters (laser power, scanning speed) on the SLM formation of the Inconel 718 alloy were calculated and analyzed. Comparing the simulation and the experimentally determined solidified track widths, the two agreed well, and the results showed that, as a rule, the width increased linearly with the laser power and decreased linearly with the scanning speed.
(4) Key to the current metal additive manufacturing process is that the geometry of the workpiece has an important influence on the thermal-melt-stress evolution. To analyze the influence of the “heat transfer process-geometry-stress distribution” on the quality of the workpiece using the molten pool dynamics model, the thermal load under different process parameters must be obtained based on the model discussed here and be introduced into the stress calculation of the workpiece in a reasonable way.
(5) The complex thermophysical interactions existing in the SLM process often occur in a very short period of time and on a microscopic scale, such that the microstructure of the workpiece is greatly affected by the SLM process. Therefore, predicting the evolution behavior of an SLM solidification structure under different process parameters is also an important direction to study the “microstructure-molten pool-performance” of SLM parts.

## Figures and Tables

**Table 1:** Physical parameters of the Inconel 718 alloy.

Parameter	Value
Density, kg/m^3^	8250 (298 K) − 7488 (1373 K) − 7803 (1638 K) − 7378 (2000 K) − 6470 (2773 K)
Solidus temperature, K	1373
Liquidus temperature, K	1638
Vaporization temperature, K	3000
Latent heat of melting, J/kg	2.19 × 10^5^
Latent heat of vaporization, J/kg	7.34 × 10^6^
Specific heat capacity, J/(kg·K)	760
Surface tension coefficient with temperature change rate, N/(m·K)	−3.24 × 10^−4^
Molecular mass, kg	9.9134 × 10^−26^
Thermal conductivity, W/(m·K)	11.03 (298 K) − 28.01 (1373 K) − 27.86 (1638 K) − 45.72 (2773 K)
Dynamic viscosity, Pa·s	0.021 (1373 K) − 0.009 (1638 K) − 0.005 (1933 K) − 0.002 (2773 K)

The constant specific heat capacity was chosen to improve the computational efficiency and the temperature dependent values were set using a simple linear interpolation.

**Table 2:** Other required calculation parameters.

Parameter	Value
Initial porosity of the powder layer [36]	0.4
Laser absorption rate [33]	0.36
Laser spot diameter, m	1.0 × 10^−4^
Average particle diameter, m	3.0 × 10^−5^
Powder bed thickness, m	4.0 × 10^−5^
Laser power, W	285
Scanning speed, m/s	0.96
Density of the base plate, kg/m^3^	7200
Thermal conductivity of the base plate, W/(m·K)	28
Specific heat capacity of the base plate, J/(kg·K)	640
Density of the gas phase, kg/m^3^	1.225
Thermal conductivity of the gas phase, W/(m·K)	0.0242
Specific heat capacity of the gas phase, J/(kg·K)	1006.43
Convective/radiation heat transfer temperature of the surroundings, K	300
Convective heat transfer coefficient of the lower surface of the base plate and the upper surface of the powder layer, W/(m^2^·K)	80
Emissivity	0.36
Initial temperature, K	353.15
Stefan–Boltzmann constant, W/(m^2^·K^4^)	5.67 × 10^−8^
Boltzmann constant, J/K	1.3806505(24) × 10^−23^
Standard atmospheric pressure, Pa	1.01325 × 10^5^

**Table 3:** Related parameters of this experiment.

Parameter	Value
Laser spot diameter, m	7.0 × 10^−5^
Powder bed thickness, m	3.0 × 10^−5^
Laser power, W	150, 200, 250, 300, 350
Scanning speed, m/s	0.4, 0.5, 0.6, 0.7, 0.8
